# HDAC6, A Novel Cargo for Autophagic Clearance of Stress Granules, Mediates the Repression of the Type I Interferon Response During Coxsackievirus A16 Infection

**DOI:** 10.3389/fmicb.2020.00078

**Published:** 2020-01-31

**Authors:** Yingcheng Zheng, Guoguo Zhu, Yinglian Tang, Jun Yan, Song Han, Jun Yin, Biwen Peng, Xiaohua He, Wanhong Liu

**Affiliations:** ^1^Hubei Province Key Laboratory of Allergy and Immunology, Department of Immunology, School of Basic Medical Sciences, Wuhan University, Wuhan, China; ^2^Shenzhen Research Institute, Wuhan University, Shenzhen, China; ^3^Department of Emergency, General Hospital of Central Theater Command of People’s Liberation Army of China, Wuhan, China

**Keywords:** autophagy, stress granule, HDAC6, coxsackievirus A16, type I interferon response

## Abstract

Autophagic cargoes ensure selective autophagy for the recognition and removal of various cytosolic aggregated proteins, damaged organelles, or pathogens. Stress granules (SGs), as antiviral immune complexes, serve a positive role in the type I interferon (IFN) response and can be targeted by autophagy (termed granulophagy). However, the cargo of granulophagy remains elusive, and it is still unknown whether granulophagy plays a role in viral infection. Here, we found that histone deacetylase 6 (HDAC6), a component of viral RNA-induced SGs, is a novel granulophagic cargo that is recognized by p62/Sequestosome 1 (SQSTM1) and mediates the degradation of SGs in coxsackievirus A16 (CA16)-infected cells. CA16 viral RNA activated the protein kinase RNA-activated (PKR)/eukaryotic translation initiation factor 2-alpha (eIF2α) pathway to promote SG assembly. The SGs were degraded by CA16-triggered autophagy via the interaction between the ubiquitin-associated (UBA) domain of p62 and the ubiquitin-binding domain (UBD) of HDAC6, which was bridged by a poly-ubiquitin chain. We also found that granulophagy repressed the type I interferon response and facilitated viral replication. These results suggest that HDAC6 might be the first identified granulophagic cargo and granulophagy could be a strategy that viruses apply to repress the antiviral immune response.

## Introduction

Selective autophagy relies on autophagic cargo recognition to remove various cytosolic aggregated proteins, damaged organelles, or pathogens. Autophagic receptors, such as p62/Sequestosome 1 (SQSTM1), recognize autophagic cargo and, by binding to small ubiquitin-like modifiers [microtubule-associated proteins light chain 3 (MAP1LC3/LC3), autophagy-related gene 5 (Atg5), autophagy-related gene 8 (Atg8), and gamma-aminobutyric acid receptor-associated proteins (GABARAPs)], mediate the formation of selective autophagosomes and finally, lysosomal degradation (Gatica et al., 2018). SGs are large cytoplasmic foci that are nucleated by the aggregation of untranslated messenger ribonucleoproteins (mRNPs), which accumulate as a result of stress-induced translation arrest (Anderson and Kedersha, 2009). SGs are dynamic structures. They can be targeted for autophagic clearance, and this selective autophagy is termed granulophagy (Protter and Parker, 2016). A key observation is that inhibition of valosin-containing protein (VCP)/Cdc48 function in yeast or mammals leads to the accumulation of stress granules in the cytosol (Buchan et al., 2013). However, selective autophagy cargo remains elusive. Thus, the identification of autophagic cargo for granulophagy represents a rational strategy to gain insight into the molecular mechanisms that govern granulophagy.

Viruses manipulate some kinds of selective autophagy to facilitate viral replication. It has been reported that mitochondria-targeted autophagy (termed mitophagy), lipid droplet-targeted autophagy (termed lipophagy), and endoplasmic reticulum-targeted autophagy (termed reticulophagy or ERophagy) could be induced by viruses to facilitate their replication (Heaton and Randall, 2010; Ding et al., 2017; Lennemann and Coyne, 2017). However, it is still unclear whether granulophagy occurs during virus infection and its role in viral replication. There is accumulating evidence demonstrating the antiviral nature of stress granules, as SGs are platforms for sensing viral molecular patterns and initiate antiviral signaling (McCormick and Khaperskyy, 2017). Retinoic acid inducible gene-I (RIG-I)-like receptors (RLRs) detect viral non-self RNA in the cytoplasm and mediate the production of antiviral cytokines, such as IFN-β (Yoneyama et al., 2016). The eIF2α kinase PKR is the primary sensor responsible for the rapid inhibition of translation initiated by dsRNA (Garcia et al., 2007). By phosphorylating eIF2α, PKR promotes SG assembly and induces type I IFN production (Yoneyama et al., 2016). Thus, SGs are believed to be an antiviral immune complex. Viruses apply various strategies to disrupt SG response pathways at multiple levels to facilitate self-replication. A typical virus in this case is influenza A virus. This virus encodes non-structural protein 1 (NS1) that shields viral RNA from detection by PKR and blocks antiviral SG response pathways (Talon et al., 2000). Autophagy is manipulated by viruses for their immune evasion, replication and release from infected cells (Choi et al., 2018). Viruses have the potential to apply autophagy to selectively degrade immune complexes associated with the antiviral response (Choi et al., 2018). However, it remains unclear whether SG-targeted autophagy would also lead to repression of the antiviral response. Thus, confirmation of granulophagy occurring during virus infection would provide a deeper insight into the antiviral immune response.

Previously, we focused on the molecular epidemiology and pathogenic mechanism of CA16 and found that CA16 infection induces apoptosis (Zhu et al., 2013) and regulates long non-coding RNA (lncRNA) (Shi et al., 2016), which regulates viral replication (Zhu et al., 2013; Shi et al., 2016). In this study, we found that granulophagy occurred during CA16 infection and facilitated viral replication, and CA16-induced granulophagy also repressed the antiviral response. Moreover, we demonstrated that HDAC6 could be a granulophagy cargo.

## Materials and Methods

### Cells, Virus and Plasmids

Rhabdomyosarcoma (RD) cells (ATCC, CCL-136) were maintained in modified Eagle’s medium supplemented with 10% heat-inactivated fetal bovine serum (FBS, Biological Industries, Lot No. 1719426) and penicillin (100 U/ml)/streptomycin (100 μg/ml) (BioSharp, Cat No. BL505A) at 37°C in a humidified atmosphere containing 5% CO_2_.

The CA16 strain (obtained from the China Center for Type Culture Collection) was prepared by infecting RD cells at a multiplicity of infection (MOI) of 0.1 and harvesting culture fluid at 48 hpi. Clarified culture fluid was aliquoted and stored at −80°C. Virus infectivity was assessed by plaque assay on RD cell monolayers.

shDNAs encoding specific shRNAs targeting Atg5, Atg7, and p62 were cloned into pGPU6 (GenePharma). A negative control vector was constructed with a nonsense shRNA sequence. All inserted sequences were verified by DNA sequencing. The sequences of the shRNAs are shown in Table 1.

**TABLE 1:** Insert sequences for each target gene.

ID	Insert Sequence
p62	CCGGCGAGGAATTGACAATGGCCATCTCGAG ATGGCCATTGTCAATTCCTCGTTTTT
Atg5	CCGGCCTGAACAGAATCATCCTTAACTC GAGTTAAGGATGATTCTGTTCAGGTTTTTTG
Atg7	CCGGGCCTGCTGAGGAGCTCTCCATCTC GAGATGGAGAGCTCCTCAGCAGGCTTTTT

Wild-type and mutant human p62 and HDAC6 genes were cloned into pEGFP-N1 for fluorescence microscopy. The genes encoding viral protein 2A, 2B, 2C, 3AB, 3C, and 3D were cloned into pCMV-HA.

### Antibodies and Reagents

Mouse anti-G3BP1 (Proteintech, Cat. No. 66486-1-Ig), rabbit anti-G3BP1 (Proteintech, Cat. No. 13057-2-AP), rabbit anti-TIA1 (Proteintech, Cat. No. 12133-2-AP), rabbit anti-HDAC6 (Proteintech, Cat. No. 12834-1-AP), rabbit anti-p62 (Proteintech, Cat. No. 18420-1-AP), rabbit anti-LC3 (Proteintech, Cat. No. 18725-1-AP), and mouse anti-ubiquitin (MBL, Cat. No. D071-3) were used for indirect immunofluorescence microscopy at a dilution of 1:200 and 1:1,000 for Western blotting. Rabbit anti-PKR (Proteintech, Cat. No. 18244-1-AP), rabbit anti-phosphorylated PKR (T451) (Abcam, Cat. No. ab81303), rabbit anti-eIF2α (Proteintech, Cat. No. 11170-1-AP), rabbit anti-ubiquitin (Proteintech, Cat. No. 10201-2-AP), and rabbit anti-phosphorylated eIF2α (S51) (Cell Signaling Technology Cat. No. 3398) were used at a dilution of 1:1000 for Western blotting. Horseradish peroxidase-conjugated secondary antibodies were purchased from Proteintech and used at 1:5,000. Cy3-conjugated goat anti-mouse secondary antibody (Cat. No. SA00009-1), Cy3-conjugated goat anti-rabbit secondary antibody (Cat. No. SA00009-2), FITC-conjugated goat anti-mouse secondary antibody (Cat. No. SA00003-1), and FITC-conjugated goat anti-rabbit secondary antibody (Cat. No. SA00003-2) were purchased from Proteintech and used at 1:200. For immunoprecipitation of p62, rabbit anti-p62 (Proteintech, Cat. No. 18420-1-AP) was used. For immunoprecipitation of HDAC6, rabbit anti-HDAC6 (Proteintech, Cat. No. 12834-1-AP) was used. Protein A/G agarose beads (Abmart, Cat. No. A10001M) were used. Cycloheximide (Cat. No. 5087390001) and polyinosinic–polycytidylic acid sodium salt (Cat. No. P0913) were purchased from Sigma-Aldrich.

### Immunofluorescence Staining and Confocal Microscopy

Cells were fixed for 20 min with 4% paraformaldehyde (PFA) and permeabilized for 15 min with 0.1% Triton X-100 in PBS. Premium quality normal goat serum was used as a blocking reagent to treat cells for 90 min at room temperature. Cells were incubated with the indicated primary antibodies for 10 h at 4°C, washed and incubated for 1 h with Cy3- or FITC-conjugated secondary antibodies. Following washing, the slides were mounted with a reagent containing DAPI. Images were captured by a confocal fluorescence microscope (Nikon A1R).

### Western Blotting

Cells were harvested and washed in precooled PBS and then lysed in RIPA buffer (Beyotime Technology, Cat. No. P0013B) on ice supplemented with protease inhibitor (Beyotime Technology, Cat. No. P1005) and phosphatase inhibitor cocktails (Beyotime Technology, Cat. No. P1005P1096) for 30 min. Whole cell extracts were cleared by centrifugation at 12,000 rpm for 10 min at 4°C. Total protein was quantified with a BCA protein assay kit (Thermo Fisher Scientific). Equivalent amounts of proteins were separated by 10% sodium dodecyl sulfate-polyacrylamide gel electrophoresis (SDS-PAGE) and transferred to a polyvinylidene fluoride (PVDF) membrane (Millipore) by electroblotting. Membranes were blocked with 5% evaporated milk in PBS with 0.2% Tween and were incubated with primary antibodies and peroxidase-conjugated secondary antibodies. Bound antibodies were visualized using Pierce ECL detection reagents. All western blots represent at least 3 independent experiments. All blots were probed for β-actin as a loading control.

### RNA Isolation and Real-Time PCR

Total RNA was extracted from cells using TRIzol (Invitrogen) according to the manufacturer’s instructions. Reverse transcription was performed using 400 ng of the purified RNA samples as a template (Thermo Fisher Scientific). The obtained cDNA samples were subjected to real-time PCR (Bio-Rad iQ5) by using a SYBR Green PCR Kit (TsingKe). Primer sequences are shown in Table 2. All samples were run in triplicate, and data analysis was performed using the 2^–ΔΔCt^ method.

**TABLE 2:** Primer sequences for each target gene.

ID	Primer Sequence
*Optn* forward primer	GCGGCAGGAACTTCTGCAA
*Optn* reverse primer	AAACGTGTCCAGGTTTGGGT
*Nbr1* forward primer	TCGGGTTCAGGTTGCTTCTG
*Nbr1* reverse primer	ACTGCCATCTTAAGCGCTTCTT
*Ndp52* forward primer	CCTGGTGGGGTGGAAGAC
*Ndp52* reverse primer	CACCTCTCCCTGAGTGGTAAC
*Tax1bp1* forward primer	TTGGATGGAGTACTGCTCGT
*Tax1bp1* reverse primer	TCTCAATTTTCAACTTGGAATGCT
*p62* forward primer	TTGCGGAGCCTCATCTCCTC
*p62* reverse primer	CCCGTCCTCATCCTTTCTCAAG
*Gapdh* forward primer	GAAGACGGGCGGAGAGAAAC
*Gapdh* reverse primer	TACGACCAAATCCGTTGACTCC

### Immunoprecipitation

Cells were collected and lysed with RIPA buffer (Beyotime Technology, Cat. No. P0013B) supplemented with protease inhibitor (Beyotime Technology, Cat. No. P0013D) on ice for 30 min and centrifuged at 12,000 rpm at 4°C for 10 min. The supernatant fractions were collected and incubated with 1 μg of the appropriate antibody at 4°C overnight and precipitated with protein A/G-agarose beads (Abmart, Cat. No. A10001) for another 4 h at 4°C. The beads were washed with lysis buffer 3 times by centrifugation at 3,000 rpm at 4°C. The immunoprecipitated proteins were separated by SDS-PAGE and western blotting was performed as previously described.

### Luciferase Reporter Assays

RD cells were plated into 24-well dishes and transfected the following day. A total of 100 ng of the reporter plasmid containing the IFN-β promoter, 1 ng of the *Renilla* luciferase control plasmid (pRL-TK), and the indicated amounts of the expression plasmids were transfected per well. The cells were infected with CA16 or were mock infected. Luciferase activities were then measured by using a Dual-Luciferase Reporter Assay System (Promega, Cat. No. E1980) according to the manufacturer’s instructions. Firefly luciferase activity was normalized to *Renilla* luciferase activity. Finally, the relative luciferase activities were expressed as fold changes over the empty-plasmid-transfected or mock controls.

### Virus Titration

Virus titers were determined by endpoint titration according to the method of Reed and Muench and expressed as 50% cell culture infective dose (CCID50). Briefly, RD cells were seeded in a 96-well plate 24 h before virus infection. Virus supernatants were serially diluted, and 100 μl of the prepared solutions was added to each well in replicates of eight. Six days after infection, the 50% cell culture infection dose (CCID50) was calculated by the Reed-Muench method.

### Statistical Analysis

The sample size for each experiment is included in the associated figure legend. All experiments were performed in three independent biological replicates. Statistical significance was determined by Student’s *t* test and one-way ANOVA followed by Tukey’s test using GraphPad Prism 7.0. For comparison in which multiple variables were tested for multiple groups, two-way ANOVA was performed. *P* values < 0.05 were considered significant. Asterisk coding is as follows: ^∗^*p* < 0.05; ^∗∗^*p* ≤ 0.01; ^∗∗∗^*p* ≤ 0.001. Data with error bars depict the average with the SEM or SD as described in the figure legends.

## Results

### SGs Are Targeted by Autophagy in CA16-Infected Cells

**FIGURE 1:**
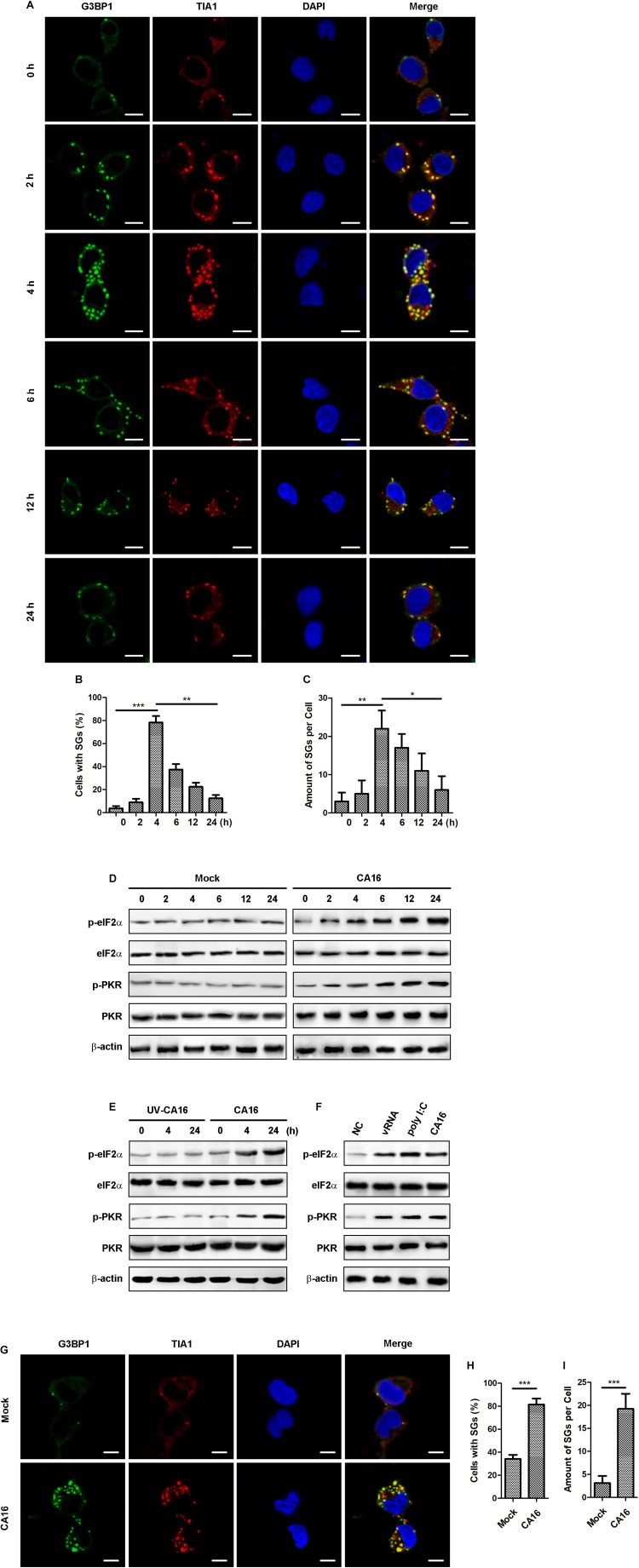
CA16 regulated the stress granule response. **(A)** RD cells were infected with CA16 at an MOI of 1 for the indicated time. SGs were examined by fluorescence microscopy (G3BP1 and TIA1 serve as SG markers). Representative images of stress granules are shown. Scale bars, 5 μm. **(B)** and **(C)** Quantitation of the data in **(A)**. Graphs show the mean ± SEM, 6 random fields and 10 cells per field were examined for confocal microscopy. **(D)** and **(E)** RD cells were infected with CA16 at an MOI of 1, UV-inactivated CA16, or were subjected to mock infection for the indicated times. **(F)** RD cells were transfected with viral RNA from CA16 (2 μg/ml), poly I:C (2 μg/ml), or were infected with CA16 at an MOI of 1 for 6 h. **(D–F)** Cell lysates were immunoblotted for anti-phosphorylated eIF2α, anti-eIF2α, anti-phosphorylated PKR, anti-PKR, and anti-β-actin. The data are representative of three independent experiments. **(G)** RD cells were subjected to CA16 infection at an MOI of 1 or mock infection for 4 h. SGs were examined by fluorescence microscopy (G3BP1 and TIA1 serve as SG markers). Representative images of stress granules are shown. Scale bars, 5 μm. **(H)** and **(I)** Quantitation of the data in **(G)**. Graphs show the mean ± SEM, 6 random fields and 10 cells per field were examined for confocal microscopy. *p < 0.05; **p < 0.01; ***p < 0.001.

Accumulating evidence supports that SGs have an antiviral nature (McCormick and Khaperskyy, 2017), and there have been no reports of granulophagy during viral infection; therefore, we explored whether CA16 infection could affect autophagic degradation of SGs. For this analysis, we used the CA16-susceptible rhabdomyosarcoma (RD) cell line. RD cells were infected with CA16 at an MOI of 1 at different time points up to 24 h post infection. The cells were immunostained for G3BP1 and TIA1, which are well-established SG-associated proteins used as SG markers (Zhang et al., 2018). Immunofluorescence (IF) microscopy revealed that CA16 infection promoted SG formation persistently for at least 4 h (Figures 1A–C). The amount of SGs was significantly increased in CA16-infected cells compared to mock-infected cells (Figures 1G–I). However, the number of CA16-infected cells containing SGs and the amount of SGs in CA16-infected cells was reduced at 12 h and 24 h post infection (Figure 1). Cycloheximide (CHX) is considered an efficient inhibitor of canonical SG formation and maintenance because it traps mRNAs in polysomes by blocking translational elongation (Kedersha et al., 2000). To confirm the type of SGs induced by CA16 infection, CA16-infected cells or mock-infected cells were treated with CHX. CHX treatment of virus-infected cells resulted in significant disassembly of CA16-induced SGs (Supplementary Figure S1). These results suggested that CA16 induced canonical SG formation.

Given that CA16 is a positive-strand non-enveloped RNA virus, we reasoned that CA16 caused SG formation via the PKR/eIF2α pathway because PKR is a viral RNA sensor that leads to SG assembly when it is activated and phosphorylates eIF2α. Immunoblotting assays showed that the phosphorylation levels of PKR and eIF2α persistently increased for 24 h compared to mock-infected cell and UV-inactivated CA16-infected cells (Figures 1D,E). Next, we further determined which component of CA16 induced the SGs. Exogenous expression of each viral protein did not affect SG assembly (Supplementary Figure S2). However, transfection of CA16 viral RNA led to a significant increase in the amount of SGs (Supplementary Figure S2). Viral RNA from CA16 also promoted the phosphorylation levels of PKR and eIF2α (Figure 1F). These results suggested that CA16 triggered SG assembly via viral RNA by activating the PKR/eIF2α pathway.

**FIGURE 2:**
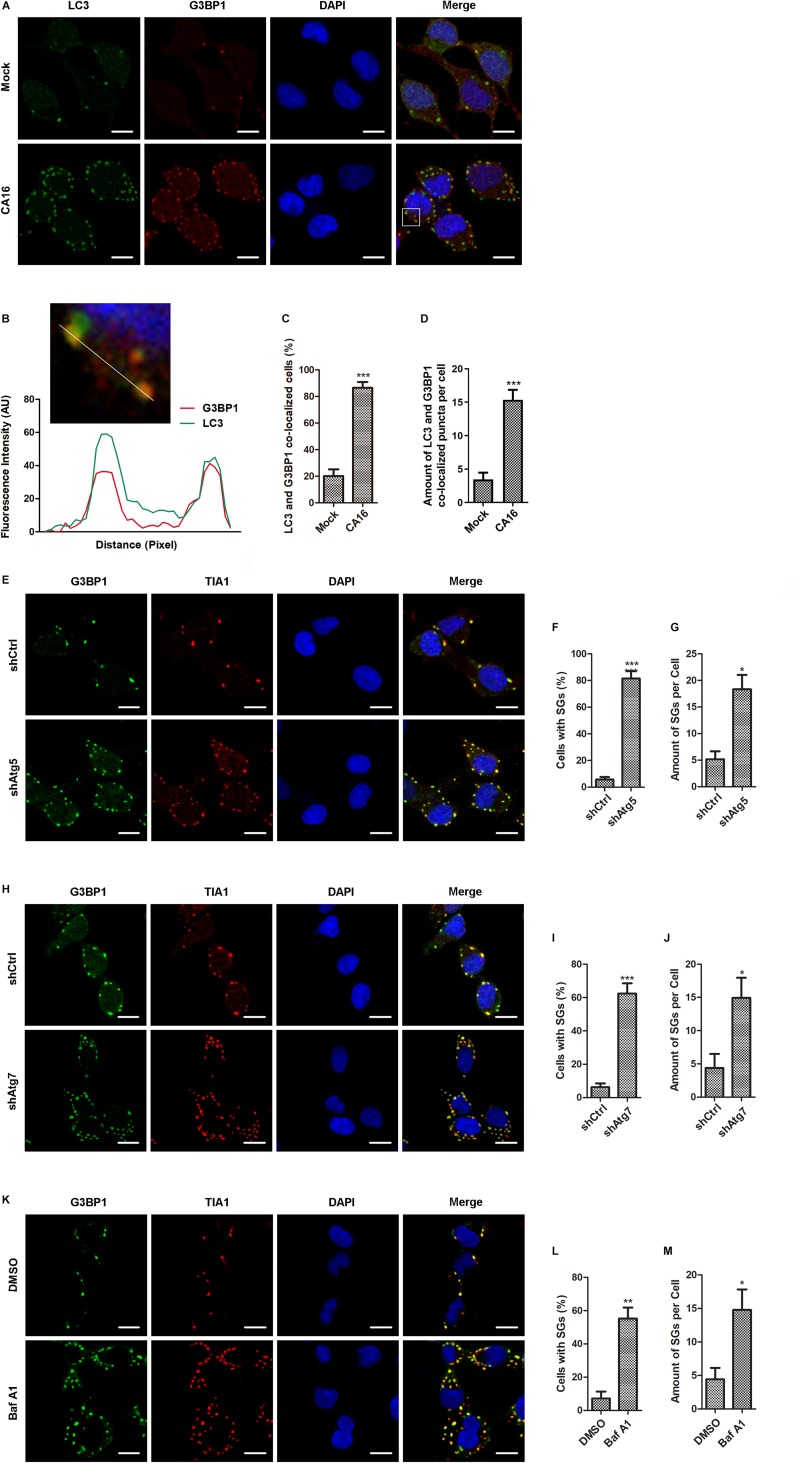
CA16-induced SGs were targeted by autophagy. **(A)** RD cells were subjected to CA16 infection at an MOI of 1 or mock infection for 4 h. SGs and autophagosomes were examined by fluorescence microscopy (G3BP1 serves as an SG marker and LC3 serves as an autophagosome marker). Representative images are shown. Scale bars, 5 μm. **(B)** The white box (inset) shows the zoomed image. The fluorescence intensity of LC3 and G3BP1 along the indicated line were scanned. Their colocalization was determined by using the Pearson correlation coefficient method (*r* = 0.71). **(C)** and **(D)** Quantitation of the data in **(A)**. Graphs show the mean ± SEM, 6 random fields and 10 cells per field were examined for confocal microscopy. **(E)** Atg5-deficient RD cells (shAtg5) or control RD cells (shCtrl) were infected with CA16 at an MOI of 1 for 24 h. **(H)** Atg7-deficient RD cells (shAtg7) or control RD cells (shCtrl) were infected with CA16 at an MOI of 1 for 24 h. **(K)** RD cells were infected with CA16 at an MOI of 1 for 24 h with or without 50 ng/ml bafilomycin A1 treatment. **(E)**, **(H)**, and **(K)** SGs were examined by fluorescence microscopy (G3BP1 and TIA1 serve as SG markers). Representative images of stress granules are shown. Scale bars, 5 μm. **(F)** and **(G)** Quantitation of the data in **(E)**. **(I)** and **(J)** Quantitation of the data in **(H)**. **(L)** and **(M)** Quantitation of the data in **(K)**. Graphs show the mean ± SEM, 6 random fields and 10 cells per field were examined for confocal microscopy. *p < 0.05; **p < 0.01; ***p < 0.001.

It was noted that the level of phosphorylated PKR and eIF2α persistently increased for 24 h, but the number of CA16-infected cells containing SGs and the amount of SGs in CA16-infected cells decreased at 12 h and 24 h post infection. This incongruous phenomenon interested us. Our previous study suggested that CA16 induced autophagy; thus, we speculated that the SG reduction was due to autophagy. First, we detected autophagic flux in CA16-infected cells. Immunoblotting assays showed that LC3 lipidation and p62 degradation increased with the infection time and MOI, suggesting that CA16 induced a complete autophagic flux (Supplementary Figures S3B,C). To investigate whether autophagy targeted SGs, we examined the colocalization of autophagosomes and SGs. Immunofluorescence microscopy showed that autophagosomes colocalized with SGs in the presence of CA16 infection (Figures 2A,B) and both percentage of LC3 and G3BP colocalized cells and the amount of LC3 and G3BP colocalized puncta per cell were obviously increased (Figures 2A,C,D). To further confirm whether CA16-induced autophagy participates in SG degradation, two key Atgs involved in autophagosome formation were ablated to inhibit autophagy initiation. As shown in Figure 2, ablation of Atg5 (Figures 2E–G) and Atg7 (Figures 2H–J) had a pronounced effect on the accumulation of SGs in CA16-infected cells. Accordingly, lysosome inhibition also promoted the accumulation of SGs in CA16-infected cells (Figures 2K–M). These findings suggested that CA16-induced SG clearance was dependent on autophagy.

### CA16-Triggered Autophagy Participates in the Clearance of Stress Granules

**FIGURE 3:**
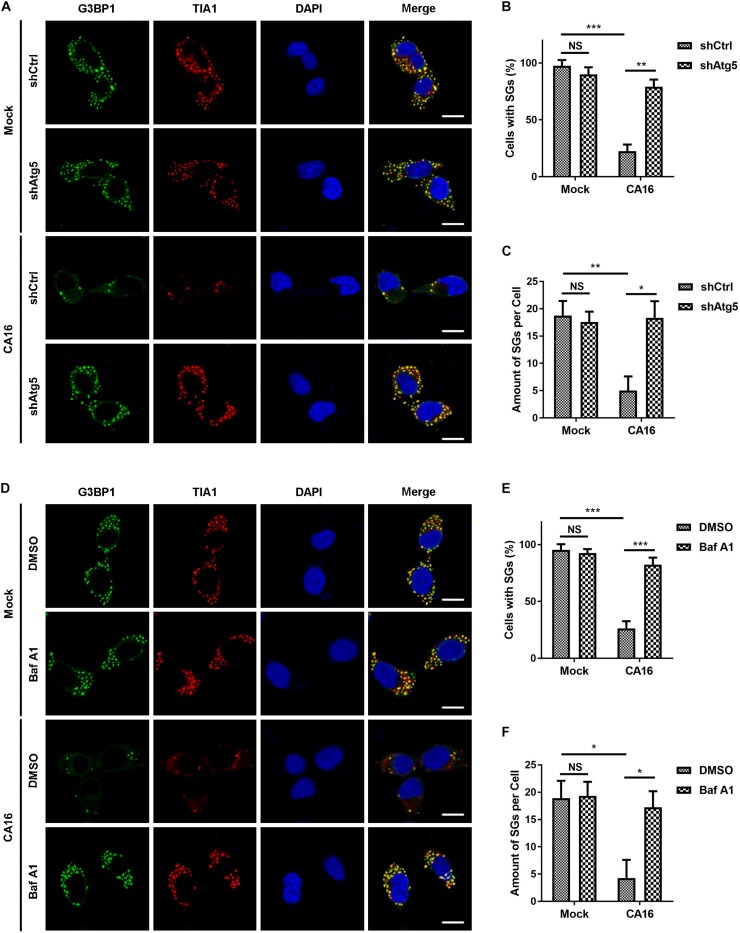
CA16-induced autophagy promoted SG clearance. **(A)** Atg5-deficient RD cells (shAtg5) or control RD cells (shCtrl) were infected with CA16 at an MOI of 1 for 24 h or were mock infected. All cells were treated with 2 μg/ml poly I:C every 6 h. SGs were examined by fluorescence microscopy (G3BP1 and TIA1 serve as SG markers). Representative images of stress granules are shown. Scale bars, 10 μm. **(B)** and **(C)** Quantitation of the data in **(A)**. **(D)** RD cells treated with 50 nM bafilomycin A1 or DMSO were infected with CA16 at an MOI of 1 for 24 h or were mock infected. All cells were treated with poly I:C 2 μg/ml every 6 h. SGs were examined by fluorescence microscopy (G3BP1 and TIA1 serve as SG markers). Representative images of stress granules are shown. Scale bars, 10 μm. **(E)** and **(F)** Quantitation of the data in **(D)**. Graphs show the mean ± SEM, 6 random fields and 10 cells per field were examined for confocal microscopy. *p < 0.05; **p < 0.01; ***p < 0.001.

SGs serve positive roles in antiviral immunity by activating the IFN-I response pathway (McCormick and Khaperskyy, 2017). Polyinosinic-polycytidylic acid (poly I:C) is a synthetic viral RNA mimic and is also an SG inducer that can trigger the SG-associated IFN-I response. Next, we investigated the effects of CA16-induced autophagy on the SG-associated IFN-I response. As expected, poly I:C induced SG assembly in RD cells (Supplementary Figure S2). CA16 infection significantly reduced the number of poly I:C-induced SGs, and knockdown of Atg5 (Figures 3A–C) rescued the degradation of the poly I:C-induced SGs, which was caused by CA16 infection. Accordingly, inhibition of the lysosome also caused a significant increase in poly I:C-induced SG accumulation (Figures 3D–F). These data suggested that CA16 repressed SG accumulation, which was closely related to autophagy. Poly I:C and CA16 could induce apoptosis (Zhu et al., 2013; Bianchi et al., 2017). To investigate whether poly I:C or CA16 induced apoptosis to promote the SGs-targeted autophagy, we applied Z-VAD-FMK, an apoptosis inhibitor, to repress apoptosis to investigate degradation of poly I:C-induced SGs which caused by CA16. Z-VAD-FMK treatment did not affect the CA16-induced SGs degradation. These data suggested that neither poly I:C- nor CA16-triggered apoptosis participates CA16-induced granulophagy (Supplementary Figure S6).

### p62 Participates in the Autophagic Clearance of SGs in CA16-Infected Cells

**FIGURE 4:**
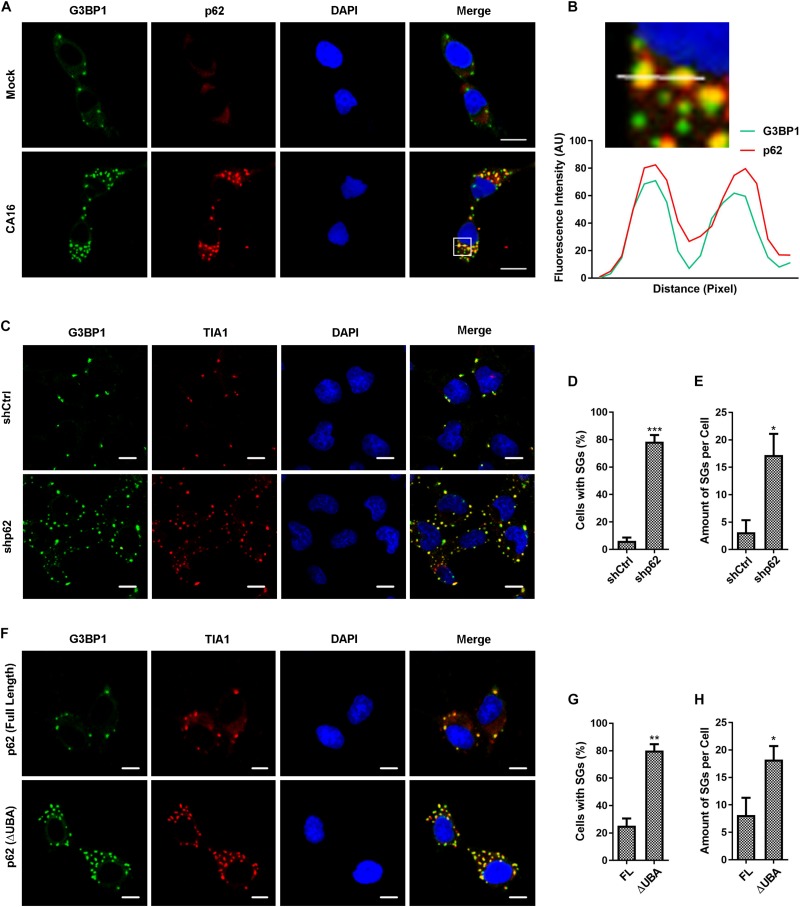
p62 mediated autophagic clearance of SGs in CA16-infected cells. **(A)** RD cells were subjected to CA16 infection at an MOI of 1 or mock infection for 4 h. The dissemination of G3PB1 and p62 was detected by fluorescence microscopy. Representative images are shown. Scale bars, 5 μm. **(B)** The white box (inset) shows the zoomed image. The fluorescence intensity of G3BP1 and p62 along the indicated line were scanned. Their colocalization was determined by using the Pearson correlation coefficient method (*r* = 0.83). **(C)** p62-deficient RD cells (shp62) or control RD cells (shCtrl) were infected with CA16 at an MOI of 1 for 24 h. **(F)** p62-deficient RD cells transduced with full-length human p62 (FL) or p62 (ΔUBA, Δ404aa-425aa) constructs were subjected to CA16 infection at an MOI of 1 for 24 h. **(C)** and **(F)** SGs were examined by fluorescence microscopy (G3BP1 and TIA1 serve as SG markers). Representative images of stress granules are shown. Scale bars, 5 μm. **(D)** and **(E)** Quantitation of the data in **(C)**. **(G)** and **(H)** Quantitation of the data in **(F)**. Graphs show the mean ± SEM, 6 random fields and 10 cells per field were examined for confocal microscopy. *p < 0.05; **p < 0.01; ***p < 0.001.

We screened changes in autophagic receptor transcription in CA16-infected cells and found that there was a significant increase in p62 mRNA levels in CA16-infected cells over time (Supplementary Figure S3A). These results suggest that p62 is a key autophagic receptor of CA16-induced autophagy. To confirm this, we investigated the dissemination of p62 and G3BP1 in CA16-infected cells. As shown in Figures 4A,B, p62-containing aggregates colocalized or were adjacent with SGs. To further confirm that p62 participates in CA16-triggered granulophagy, we investigated the effects of p62 deficiency on the amount of SGs that were induced by CA16 infection. p62-deficient macrophages exhibited enhanced accumulation of SGs after CA16 infection (Figures 4C–E). Deletion of the ubiquitin-associated domain (UBA) leads to p62 dysfunction involving autophagic cargo recognition, which leads to a deficiency in p62-mediated autophagy (Komatsu et al., 2012). As expected, UBA deletion resulted in an increase in the accumulation of SGs in CA16-infected cells (Figures 4F–H). These results suggested that the autophagic receptor p62 mediated the granulophagy process.

### HDAC6 Is a Component of CA16-Induced SGs and Serves as Autophagic Cargo

**FIGURE 5:**
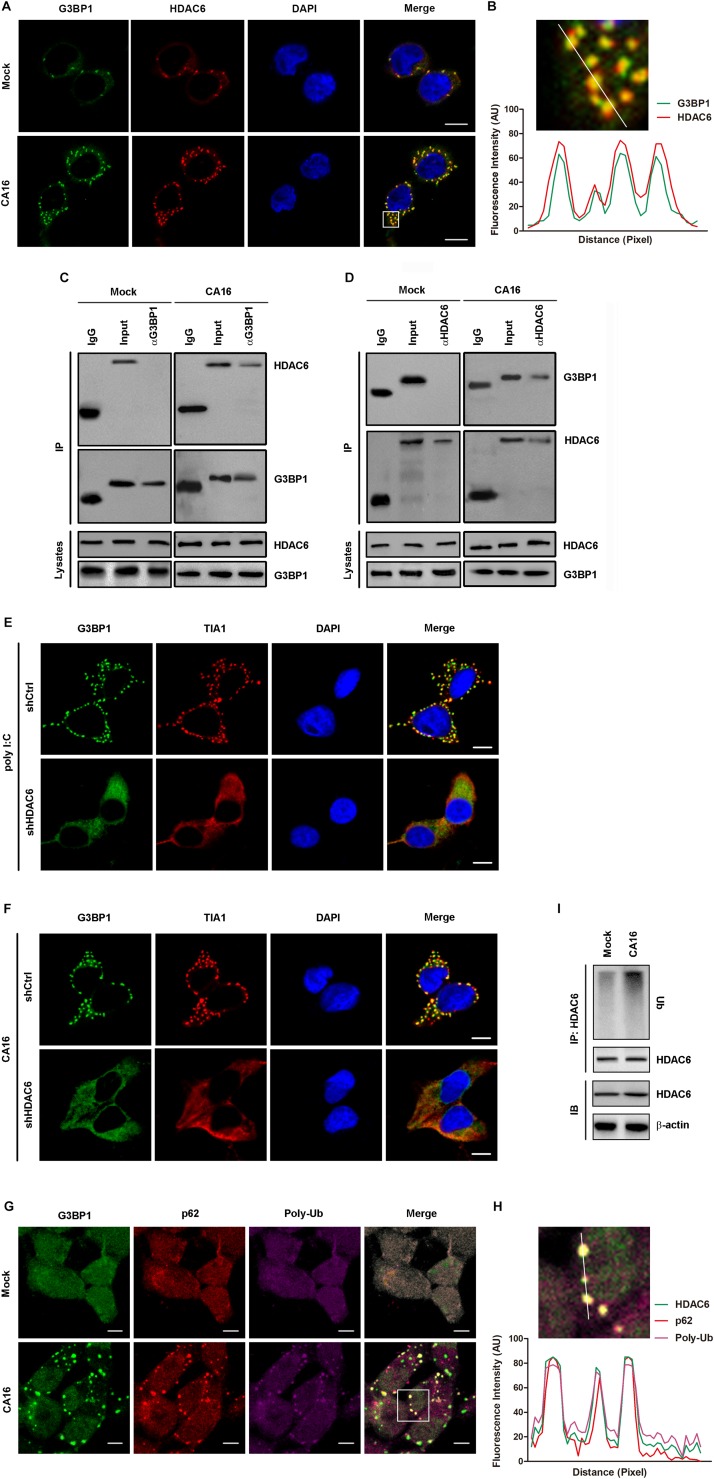
The CA16-induced SG component HDAC6 served as an autophagic cargo. **(A)** RD cells were subjected to CA16 infection at an MOI of 1 or mock infection for 4 h. The dissemination of G3PB1 and HDAC6 was detected by fluorescence microscopy. Representative images are shown. Scale bars, 5 μm. **(B)** The white box (inset) shows the zoomed image. The fluorescence intensity of G3BP1 and HDAC6 along the indicated line were scanned. Their colocalization was determined by using the Pearson correlation coefficient method (*r* = 0.64). **(C)** and **(D)** RD cells were infected with CA16 at an MOI of 1 for 4 h or with mock infection. Lysates were immunoprecipitated with **(C)** anti-G3BP1 or **(D)** anti-HDAC6 antibody. Immune complexes were resolved by SDS–PAGE and immunoblotted with anti-HDAC6 and anti-G3BP1 antibodies. The data are representative of three independent experiments. **(E)** HDAC6-deficient RD cells (shHDAC6) or control RD cells (shCtrl) were treated with poly I:C 2 μg/ml or **(F)** were infected with CA16 at an MOI of 1 for 4 h or with mock infection. SGs were examined by fluorescence microscopy (G3BP1 and TIA1 serve as SG markers). Representative images of stress granules are shown. Scale bars, 5 μm. **(G)** and **(I)** RD cells were infected with CA16 at an MOI of 1 for 4 h or with mock infection. The dissemination of G3PB1, p62 and poly ubiquitin (Poly-Ub) was detected by fluorescence microscopy. Representative images are shown. Scale bars, 5 μm. **(H)** The white box (inset) shows the zoomed image. The fluorescence intensity of G3PB1, p62, and Poly-Ub along the indicated line were scanned. Their colocalization was determined by using the Pearson correlation coefficient method (*r* = 0.71). **(I)**. The lysates were immunoprecipitated using HDAC6 antibody and blotted with the indicated antibodies. The data are representative of three independent experiments.

It has been reported that HDAC6 is a component of SGs (Kwon et al., 2007) and is also a cargo for selective autophagy (such as aggrephagy and mitophagy) (Rogov et al., 2014); thus, this molecule could be a mediator of granulophagy. To verify whether it is in the case of CA16-induced granulophagy, we first investigated whether HDAC6 is a component of CA16-induced stress granules. Immunofluorescence assay on the cellular distribution of HDAC6 showed that HDAC6 aggregates colocalized with SGs (Figures 5A,B). HDAC6 immunoprecipitated with G3BP1, which is an essential component of SGs (Figures 5C,D). To further confirm whether HDAC6 is critical for CA16-induced SGs, we investigate poly I:C- and CA16-induced SGs assembly in HDAC6 knockdown cells. There is no canonical SGs in HDAC6 knockdown cells with the treatment of poly I:C or CA16 infection (Figures 5E,F). These data suggested that HDAC6 is essential for poly I:C and CA16-induced SGs assembly. To confirm whether HDAC6 is the ubiquitinated cargo that is recognized by p62, we next investigated the distribution of HDAC6, p62, and ubiquitin chain (poly-Ub) aggregates. CA16 infection induced the formation of poly-Ub aggregates, most of which colocalized with SGs and p62 aggregates (Figures 5G,H), and the ubiquitination level of HDAC6 significantly increased in CA16-infected cells (Figure 5I). These results show that HDAC6 is a component of CA16-induced SGs and plays a role as an autophagic cargo to mediate CA16-induced granulophagy.

### Ubiquitin-Binding Domain (UBD) of HDAC6 Is Required for p62 Recognition and Autophagic Clearance of SGs in CA16-Infected Cells

**FIGURE 6:**
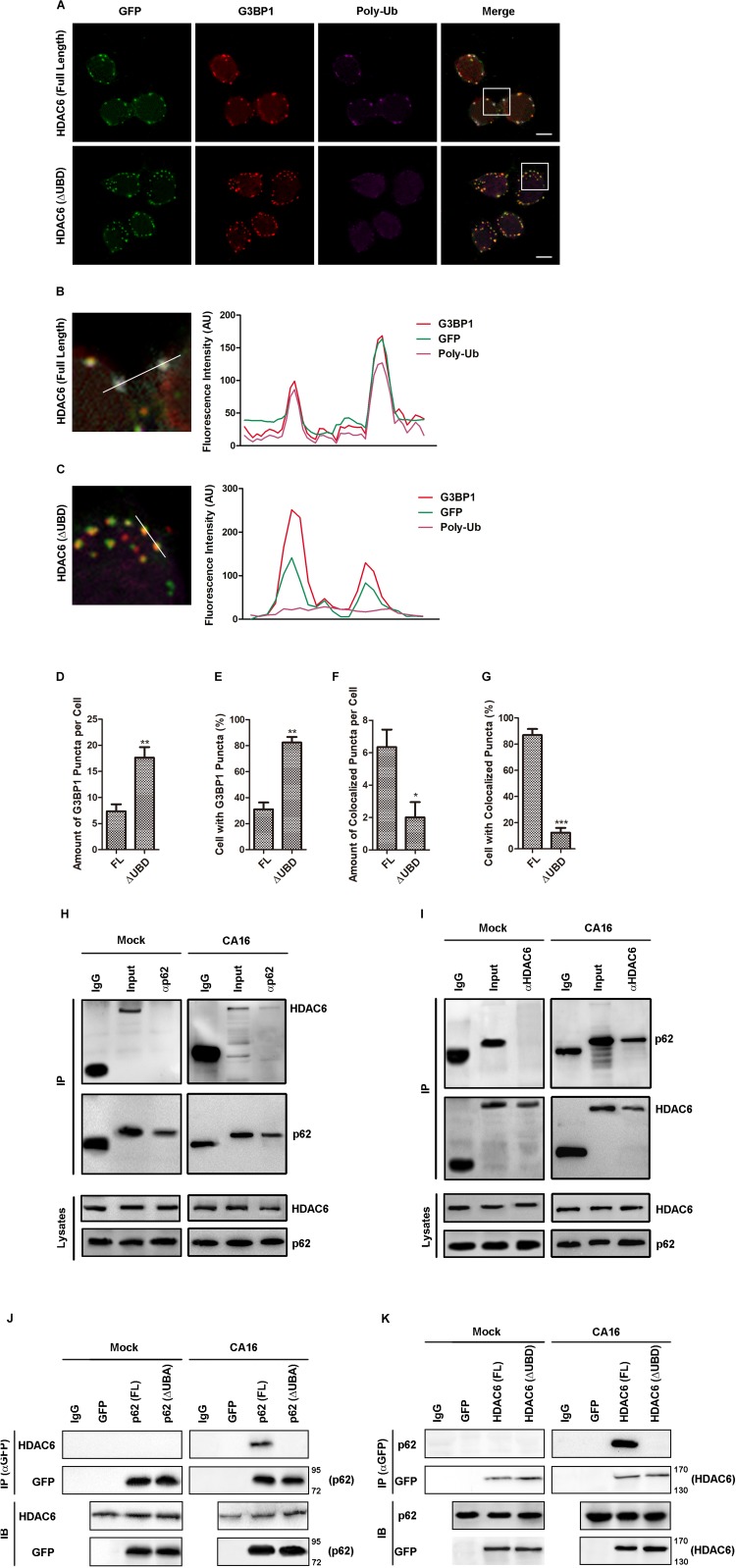
The UB domain of HDAC6 was critical for p62-mediated autophagic clearance of SGs in CA16-infected cells. **(A)** RD cells expressing GFP-HDAC6 [HDAC6 (full length)] or GFP-HDAC6 with ubiquitin binding domain deletion [HDAC6 (ΔUBD, Δ1154aa-1189aa)] were subjected to CA16 infection at an MOI of 1 for 24 h. The intracellular distribution of G3BP1 and GFP was examined by confocal microscopy. Scale bars, 5 μm. **(B)** and **(C)** The white box (inset) shows the zoomed image. The fluorescence intensity of G3BP1, GFP, and poly-Ub along the indicated line were scanned. **(D–G)** Quantitation of the data in **(F)**. Graphs show the mean ± SEM, 6 random fields and 10 cells per field were examined for confocal microscopy. **(H)** and **(I)** RD cells were infected with CA16 at an MOI of 1 for 4 h. Lysates were immunoprecipitated with **(H)** anti-p62 or **(I)** anti-HDAC6 antibody. Immune complexes were resolved by SDS–PAGE and immunoblotted with anti-HDAC6 and anti-p62 antibodies. The data are representative of three independent experiments. **(J)** RD cells expressing GFP-p62 [p62 (full length)] or GFP-p62 with ubiquitin-associated domain deletion [p62 (ΔUBA, Δ404aa-425aa)] were subjected to CA16 infection at an MOI of 1 for 4 h. Lysates were immunoprecipitated with anti-GFP antibody. Immune complexes were resolved by SDS–PAGE and immunoblotted with anti-HDAC6 and anti-GFP antibodies. **(K)** RD cells expressing GFP-HDAC6 [HDAC6 (full length)] or GFP-HDAC6 with ubiquitin-binding domain deletion [HDAC6 (ΔUBD, Δ1154aa-1189aa)] were subjected to CA16 infection at an MOI of 1 for 4 h. Immune complexes were resolved by SDS–PAGE and immunoblotted with anti-p62 and anti-GFP antibodies. *p < 0.05; **p < 0.01; ***p < 0.001.

HDAC6 is a Ub-binding protein that plays a role in aggresome formation and also affects selective autophagy. HDAC6 binds ubiquitin chains via the C-terminal ubiquitin-binding domain (UBD), which shows a preference for K63-linked ubiquitin chains. p62 directly binds to ubiquitinated protein aggregates via its UBA domain and sequesters them into inclusion bodies via its PB1 domain. SQSTM1 also interacts with phagophores via its LC3-interacting (LIR) motif (Lee and Weihl, 2017). Thus, we reasoned that p62 directly binds to ubiquitinated HDAC6 to mediate the autophagic clearance of SGs. Deleting the UBD of HDAC6 did not affect the CA16-induced or poly I:C-induced SG assembly (Supplementary Figure S4). Deleting the UBD of HDAC6 resulted in defective SG poly-Ub recruitment (Figures 6A–C). Indeed, almost no SG-associated poly-Ub was detected in HDAC6 UBD deletion-transfected cells in the presence of CA16 infection (Figures 6A–C). The amount of SGs and percentage of cells with SGs were obviously increased in HDAC6 UBD deletion-transfected cells in the presence of CA16 infection (Figures 6A,D,E) but the colocalization of SGs with poly-ubiquitin was significantly inhibited (Figures 6A,F,G). HDAC6 immunoprecipitated with p62 in the presence of CA16 infection (Figures 6H,I). However, deletion of the UBA domain of p62 or the UBD of HDAC6 led to inhibition of the interaction between them even in the presence of CA16 infection (Figures 6J,K). p62 interacts with HDAC6 and regulates deacetylase activity (Yan et al., 2013). To rule out whether the effect of CA16 infection on SGs was due to HDAC6 deacetylase activity, regulated by p62, we investigated the change in SG accumulation when HDAC6 deacetylase activity was inhibited in CA16-infected cells. HDAC6 deacetylase activity inhibition did not affect SG accumulation in the presence of CA16 infection (Supplementary Figure S5). These results suggested that p62 binds to ubiquitinated HDAC6 and mediates the autophagic clearance of SGs but this process does not involve HDAC6 deacetylase activity.

### CA16-Induced Autophagic Clearance of SGs Attenuates the IFN-I Response and Facilitates Viral Replication

**FIGURE 7:**
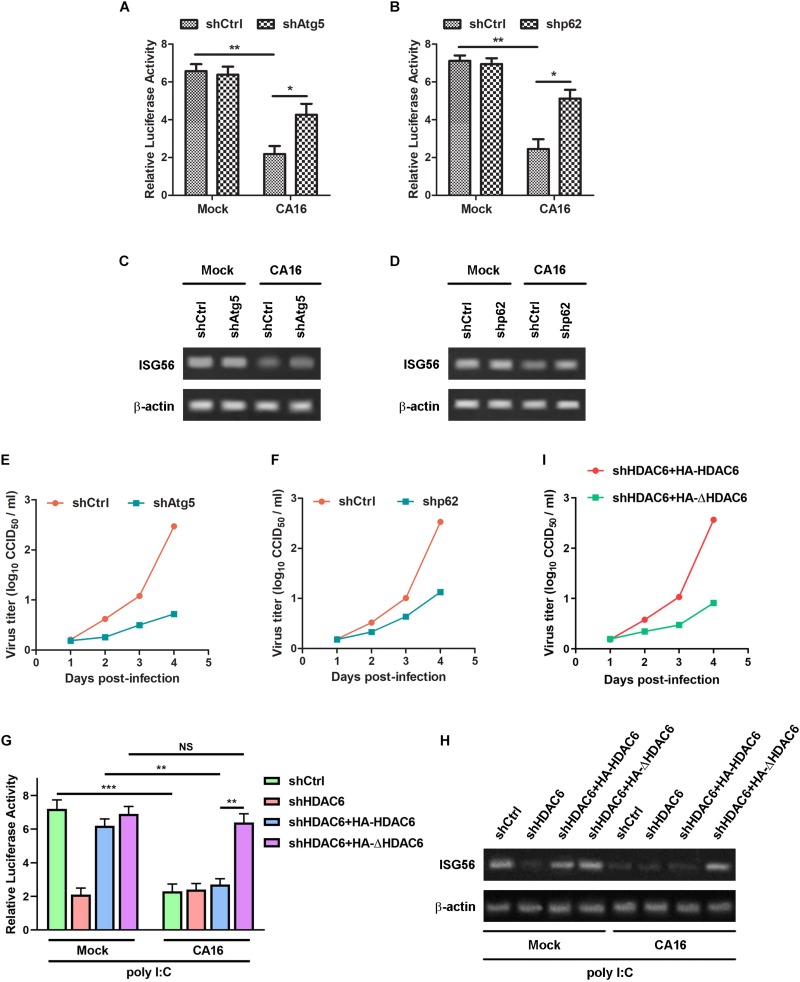
CA16-induced autophagic clearance of SGs attenuated the IFN-I response and facilitated viral replication. **(A–F)** Atg5-deficient RD cells (shAtg5), p62-deficient RD cells (shp62), or control RD cells (shCtrl) were infected with CA16 at an MOI of 1 for 24 h or were mock infected in the presence of 2 μg/ml poly I:C. **(G–I)** HDAC6-deficient RD cells (shHDAC6) were transfected HA- tagged plasmid expressing HDAC6 or UBD-deleted HDAC6 (DHDAC6, Δ1154aa-1189aa). The cells and control RD cells (shCtrl) were infected with CA16 at an MOI of 1 for 24 h or were mock infected in the presence of 2 μg/ml poly I:C. **(A,B,G)** The cells were subjected to luciferase assays. Graphs show the mean ± SD, *n* = 3. **(C,D,H)** The cells were subjected to RT-PCR assays. The data are representative of three independent experiments. **(E,F,I)** The virus titer was determined by a CCID50 assay. *p < 0.05; **p < 0.01; ***p < 0.001.

Given that SGs play a role in the IFN-I response and that CA16 induced autophagic clearance of SGs, we reasoned that CA16-induced autophagy attenuated the IFN-I response. poly I:C can not only induce the IFN response but also trigger SG assembly. Thus, it is believed to be a synthetic agonist of the SG-associated IFN response. To investigate whether CA16 infection also suppressed the SG-associated IFN-I response, we determined the CA16-induced change in IFN-β promoter activity and transcription of the IFN-β downstream gene ISG56 in poly I:C-treated cells. As expected, Atg5 (Figure 7A) or p62 (Figure 7B) deficiency led to an increase in IFN-β promoter activity (Figures 7A,B) and ISG56 transcription (Figures 7C,D) in poly I:C-treated cells in the presence of CA16 infection. Due to the antiviral nature of type I IFN, we reasoned that the CA16-induced repression of IFN-β production would result in an increase in virus replication. Accordingly, Atg5 (Figure 7E) or p62 (Figure 7F) deficiency significantly suppressed viral replication (Figures 7E,F). Since UBD of HDAC6 is essential for CA16-induced granulophagy as Figure 6 suggested, we restored WT HDAC6 or HDAC6 with UBD deletion in HDAC6 knockdown cells to investigate the role of HDAC6-mediated granulophagy in type I IFN response regulated by CA16 and viral replication. Restoration of wild type HDAC6 recovery the CA16-induced repression of IFN-β promoter activity in HDAC6 knockdown cells (Figures 7G,H) and viral replication promotion (Figure 7I) but restoration of HDAC6 with UBD deletion could not lead to this effect. These results suggest that CA16-induced repression of the IFN-I response is related to the autophagic clearance of SGs.

## Discussion

**FIGURE 8:**
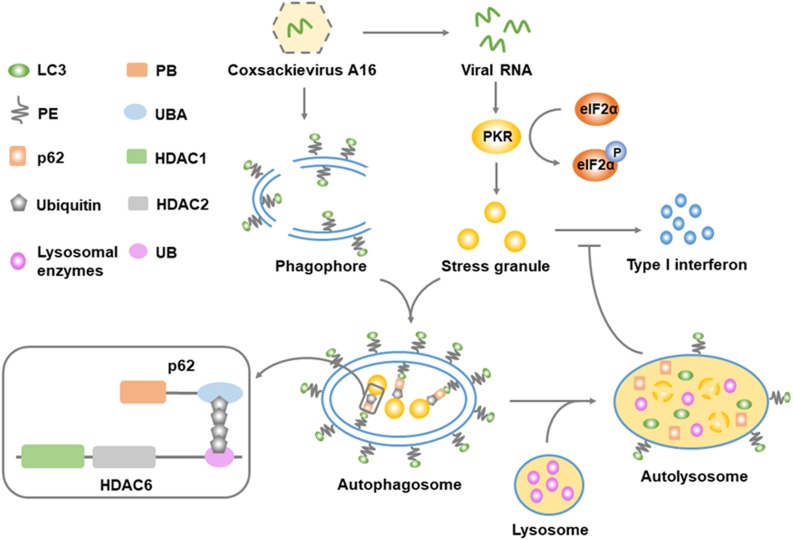
Schematic representation of the key findings. Viral RNA from CA16 activates PKR to phosphorylate eIF2α, leading to the initiation of the stress granule response. CA16-induced autophagy promotes the clearance of stress granules, which is mediated by a ubiquitin bridge between the p62 UBA domain and the HDAC6 UB domain. Autophagic clearance of stress granules results in suppression of the type I interferon response. Microtubule-associated proteins light chain 3 (LC3); phosphatidylethanolamine (PE); Phox and Bem1p domain (PB); ubiquitin-associated domain (UBA); histone deacetylase domain (HDAC); ubiquitin-binding domain (UBD).

Here, we observed that viral RNA from CA16 activated the PKR/eIF2α pathway and triggered SG assembly. CA16 induced autophagy targeted to antiviral SGs, resulting in restriction of the type I IFN response and facilitating viral replication. Moreover, we observed that HDAC6 was a component of CA16-induced SGs and interacted with p62 to anchor the SGs to the autophagosomal membrane. Ubiquitin binds to the UBD of HDAC6 and is recognized by p62. This mediated autophagic clearance of viral RNA-induced SGs (Figure 8). The data indicated that CA16 bidirectionally regulated the antiviral SG response. This the SG-targeted autophagic degradation mechanism differs from typical ways that viruses apply to repress the antiviral SG response. We also found that HDAC6 could be a cargo for granulophagy and that granulophagy was a useful tool for virus-mediated repression of the type I IFN response.

Cargo selection and receptor recognition are critical events during selective autophagy. The cargos for granulophagy, a kind of selective autophagy that targets SGs, is still unclear. SGs are assembled by untranslated mRNPs and many non-RNA-binding proteins (Jain et al., 2016). SGs are dynamic and have multiple fates. They can interact with P bodies, exchange components with the cytoplasm, and undergo autophagy (Protter and Parker, 2016). Similar to other autophagic degradation pathways, Atg protein deficiency leads to a dramatic increase in cytosolic SGs. Beyond the canonical Atg family, other mechanisms involved in the autophagic degradation of SGs, which is termed granulophagy, are still elusive. The autophagic cargo for granulophagy is one such unknown key issue. HDAC6 is a complex protein aggregate that is a critical component of SGs and is necessary for SG maturation (Reineke and Neilson, 2019). In addition, HDAC6 is a selective cargo for some types of selective autophagy, such as aggrephagy and mitophagy (Rogov et al., 2014). However, it is still unclear whether HDAC6 could be an autophagy cargo for granulophagy. We screened the canonical autophagic receptors NBR1, NDP52, OPTN, p62/SQSTM1, and TAX1BP1 in CA16-infected cells and found that the transcription level of Sqstm1 increased significantly. We also observed that CA16 promoted LC3 lipidation and p62 degradation over the infection time. This suggested that p62 could be an autophagy receptor of CA16-induced autophagy. HDAC6 has ubiquitin binding properties and serves as a cargo protein for the autophagy receptor p62. It forms aggregates of ubiquitinated proteins and anchors the aggregates to autophagosome membranes by recruiting LC3 (Nakashima et al., 2015). Our data suggested that HDAC6 might be the first identified granulophagic cargo.

Granulophagy is an important degradation pathway for SGs. Given the antiviral nature of SGs, a crucial goal is to confirm whether granulophagy can occur during viral infection and lead to the repression of the type I IFN response. Autophagy is a double-edge sword during virus infection. It is a useful tool for host cells to defend against viral infection. However, viruses are also capable of hijacking and even inducing autophagy for their benefit. It has been reported that autophagy deficiency increases IFN production in response to an RNA virus (Jounai et al., 2007). SG formation is an integral part of the antiviral response since it is a manifestation of translation arrest, which can restrain viral gene expression, and SGs are antiviral signaling platforms. Transfection of cells with poly I:C or infection of cells with some viruses, especially RNA viruses and retroviruses, leads to the recruitment of PKR, RIG-I, or MDA5 to SGs, and the respective downstream signaling cascades may proceed simultaneously. Indeed, stress granule formation, nuclear translocation of IRF3 and the transcription of type I IFNs occur in parallel (Clavarino et al., 2012). Our data indicated that either deficiency of key autophagy proteins or inhibition of critical processes in autophagic flux could lead to an increase in IFN response to the RNA virus CA16. Moreover, our data indicate that this is due to the autophagic degradation of SGs, which are critical for RIG-I-like receptor (RLR)-triggered IFN production. These data suggest that CA16 infection can induce granulophagy and that selective autophagy represses the type I IFN response.

It has been reported that some kinds of selective autophagy, such as mitophagy, lipophagy, and reticulophagy, affect viral replication. However, it is still unknown whether granulophagy affects viral replication. Selective autophagy plays important roles in viral replication. For example, human parainfluenza virus type 3 (HPIV3) matrix protein M interacts with the mitochondrial elongation factor Tu, inducing mitochondria-targeted autophagy (termed mitophagy) to inhibit the subsequent interferon response (Ding et al., 2017). Dengue virus induces selective autophagy of lipid droplets (lipophagy) to provide energy required for viral replication (Heaton and Randall, 2010). Zika virus (ZIKV) non-structural protein 4A (NS4A) and NS4B activate autophagy by inhibiting AKT and mechanistic target of rapamycin complex 1 (mTORC1) (Liang et al., 2016). Furthermore, the viral protease NS3 cleaves reticulophagy regulator 1 (FAM134B) to block reticulophagy so that ZIKV can use the ER as a replication site (Lennemann and Coyne, 2017). Our data suggested that CA16 infection could induce another selective autophagy, granulophagy, and this selective autophagy facilitated viral replication.

EVs have evolved several strategies to regulate the SG response to ensure efficient viral replication. It has been shown that EV proteinase is the major tool for repressing the SG response, but if granulophagy is one viral strategy for repressing SGs is still unclear. Enterovirus 71 (EV71) (Zhu et al., 2016), poliovirus (PV) (White and Lloyd, 2011; Fitzgerald and Semler, 2013), and coxsackievirus B3 (CVB3) (Wu et al., 2014) have the capability to induce SG formation. Poliovirus (PV) and coxsackievirus B3 (CVB3) repress SG assembly mainly by expressing proteinase to cleave SG components or SG response initiation factors (Lloyd, 2016) and have evolved several parallel strategies to regulate SGs. Most enteroviruses utilize their encoded proteinase to control or repress SGs to defend against the antiviral effects of SGs (Lloyd, 2016). Our data suggested that CA16, another enterovirus, triggered antiviral SG formation by viral RNA via the PKR/eIF2α pathway and restricted SGs by autophagy, which resulted in a decrease in the IFN response. More importantly, we found a novel mechanism of SG repression by enteroviruses, namely, that enteroviruses can repress SGs via an autophagic degradation pathway. This is also meaningful for expending knowledge involving how autophagy regulates antiviral immunity.

## Data Availability Statement

The raw data supporting the conclusions of this article will be made available by the authors, without undue reservation, to any qualified researcher.

## Author Contributions

YZ, GZ, and WL conceptualized the study. YZ, GZ, YT, and JYan worked on the data curation and formal analysis. GZ was responsible for funding acquisition, and wrote, reviewed, and edited the manuscript. YZ, GZ, YT, SH, JY, BP, and WL worked on the investigation. YZ, GZ, YT, JYan, JYin, and WL worked on the methodology. SH, JYin, BP, XH, and WL administrated the project. WL was responsible for the resources. YZ, YT, and SH were responsible for the software. GZ, XH, and WL supervised the study. YZ and WL validated the study. YZ wrote the original draft.

## Conflict of Interest

The authors declare that the research was conducted in the absence of any commercial or financial relationships that could be construed as a potential conflict of interest.

## Supplementary Material

FIGURE S1 CA16 induced canonical SG formation. **(A)** RD cells were subjected to CA16 infection at an MOI of 1 or mock infection for 24 h in the presence of 50 μg/ml CHX or DMSO (Ctrl). SGs were examined by fluorescence microscopy (G3BP1 and TIA1 serve as SG markers). Representative images of stress granules are shown. Scale bars, 5 μm. **(B)** and **(C)** Quantitation of the data in **(A)**. Graphs show the mean ± SEM, 6 random fields and 10 cells per field were examined for confocal microscopy. **p* < 0.05; ***p* < 0.01; ****p* < 0.001.

FIGURE S2 CA16 RNA induced SG assembly. **(A)** RD cells were transfected with eukaryotic expression plasmids expressing viral protein 2A, 2B, 2C, 3AB, 3C, 3D or tag HA, or were transfected with viral RNA (labeled as RNA), or were treated with poly I:C, 0.5 mM sodium arsenite (labeled as Ars) for 45 min, 1 mM sodium selenite (labeled as Se) for 2 h, or transfection reagent (labeled as TR). SGs were examined by fluorescence microscopy (G3BP1 serves as an SG marker). Representative images of stress granules are shown. Scale bars, 5 μm. **(B)** and **(C)** Quantitation of the data in **(A)**. Graphs show the mean ± SEM, 6 random fields and 10 cells per field were examined for confocal microscopy. ***p* < 0.01; ****p* < 0.001; *****p* < 0.0001.

FIGURE S3 CA16 promoted transcription of *Sqstm1* and triggered complete autophagic flux. **(A,B)** RD cells were subjected to CA16 infection at an MOI of 1 or mock infection for the indicated times. **(A)** Qualitative PCR analysis of autophagy receptors in RD cells. The results are shown as the average ± SD (*n* = 3). **(C)** RD cells were subjected to CA16 infection at the indicated MOIs or mock infection for 6 h. **(B,C)** Cell lysates were immunoblotted for anti-LC3, anti-P62, and anti-β-actin antibodies. The data are representative of three independent experiments.

FIGURE S4 UBD deletion of HDAC6 did not affect the CA16-induced SG assembly. **(A)** RD cells expressing GFP-HDAC6 [HDAC6 (full length)] or GFP-HDAC6 with ubiquitin-binding domain deletion [HDAC6 (ΔUBD)] were treated with 2 μg/ml poly I:C for 6 h **(D)** or were subjected to CA16 infection at an MOI of 1 for 4 h. Intracellular distribution of G3BP1 and GFP was examined by confocal microscopy. Scale bars, 5 μm. **(B)** and **(C)** Quantitation of the data in **(A)**. **(E)** and **(F)** Quantitation of the data in **(D)**. Graphs show the mean ± SD, 6 random fields and 10 cells per field were examined for confocal microscopy.

FIGURE S5 HDAC6 deacetylase activity did not affect CA16-induced granulophagy. **(A)** RD cells were subjected to CA16 infection at an MOI of 1 or mock infection for 24 h after pretreatment with 0.1 μM CAY10603 or DMSO for 6 h. SGs were examined by fluorescence microscopy (G3BP1 and TIA1 serve as SG markers). Representative images of stress granules are shown. Scale bars, 5 μm. **(B)** and **(C)** Quantitation of the data in **(A)**. Graphs show the mean ± SEM, 6 random fields and 10 cells per field were examined for confocal microscopy.

FIGURE S6 Apoptosis inhibition did not affect CA16-induced granulophagy. **(A)** RD cells were subjected to CA16 infection at an MOI of 1 or mock infection for 24 h with or without the treatment of Z-VAD-FMK 200 μM. SGs were examined by fluorescence microscopy (G3BP1 and TIA1 serve as SG markers). Representative images of stress granules are shown. Scale bars, 5 μm. **(B)** and **(C)** Quantitation of the data in **(A)**. Graphs show the mean ± SEM, 6 random fields and 10 cells per field were examined for confocal microscopy. ***p* < 0.01; ****p* < 0.001.
