# Supplementary figures and images for: HDAC6, A Novel Cargo for Autophagic Clearance of Stress Granules, Mediates the Repression of the Type I Interferon Response During Coxsackievirus A16 Infection
Source: Front Microbiol. 2020 Jan 31;11:78. doi: 10.3389/fmicb.2020.00078 (PMC7005486; doi:10.3389/fmicb.2020.00078)

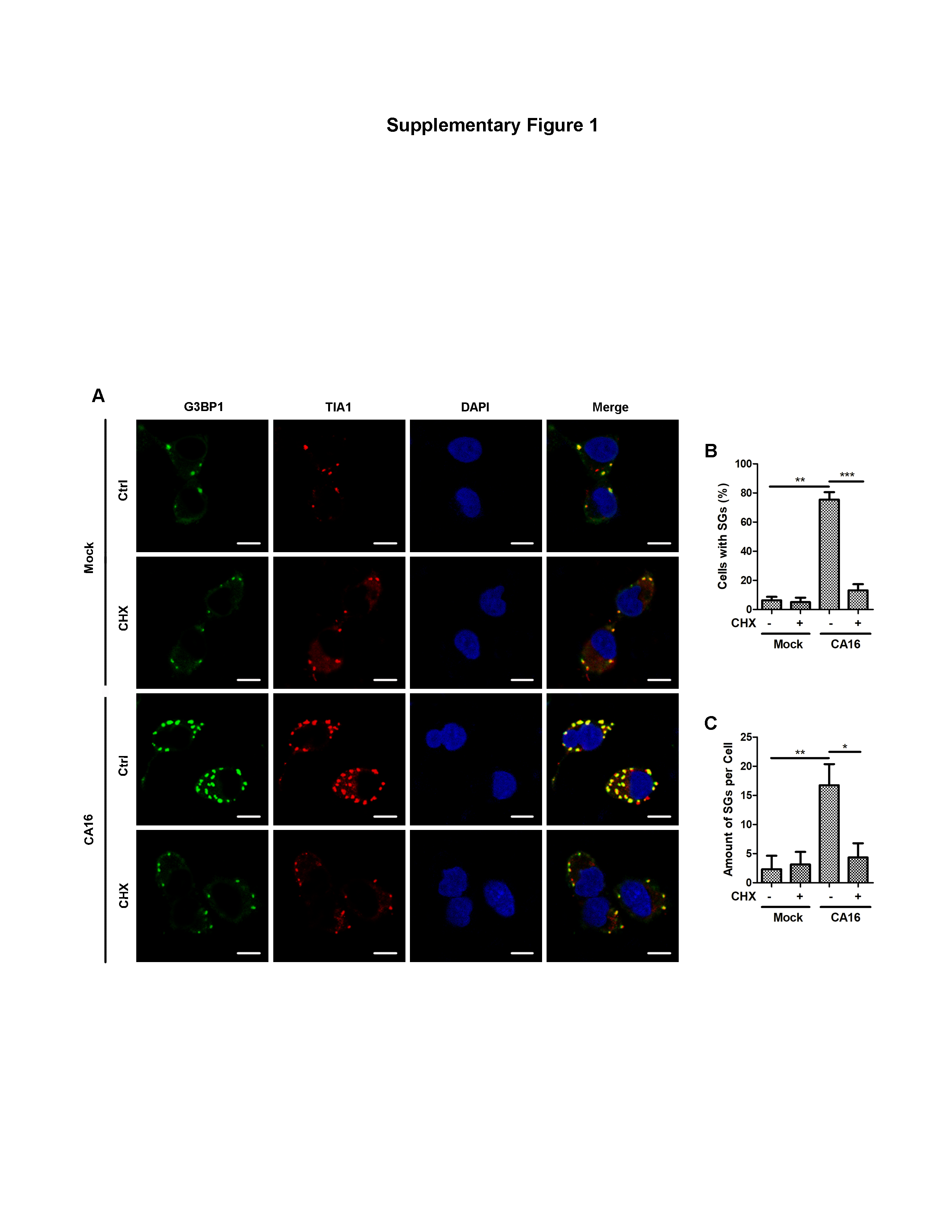

Supplement: FIGURE S1 — CA16 induced canonical SG formation. (A) RD cells were subjected to CA16 infection at an MOI of 1 or mock infection for 24 h in the presence of 50 μg/ml CHX or DMSO (Ctrl). SGs were examined by fluorescence microscopy (G3BP1 and TIA1 serve as SG markers). Representative images of stress granules are shown. Scale bars, 5 μm. (B) and (C) Quantitation of the data in (A). Graphs show the mean ± SEM, 6 random fields and 10 cells per field were examined for confocal microscopy. *p < 0.05; **p < 0.01; ***p < 0.001. [file Data_Sheet_1.zip › Image 1.TIF]

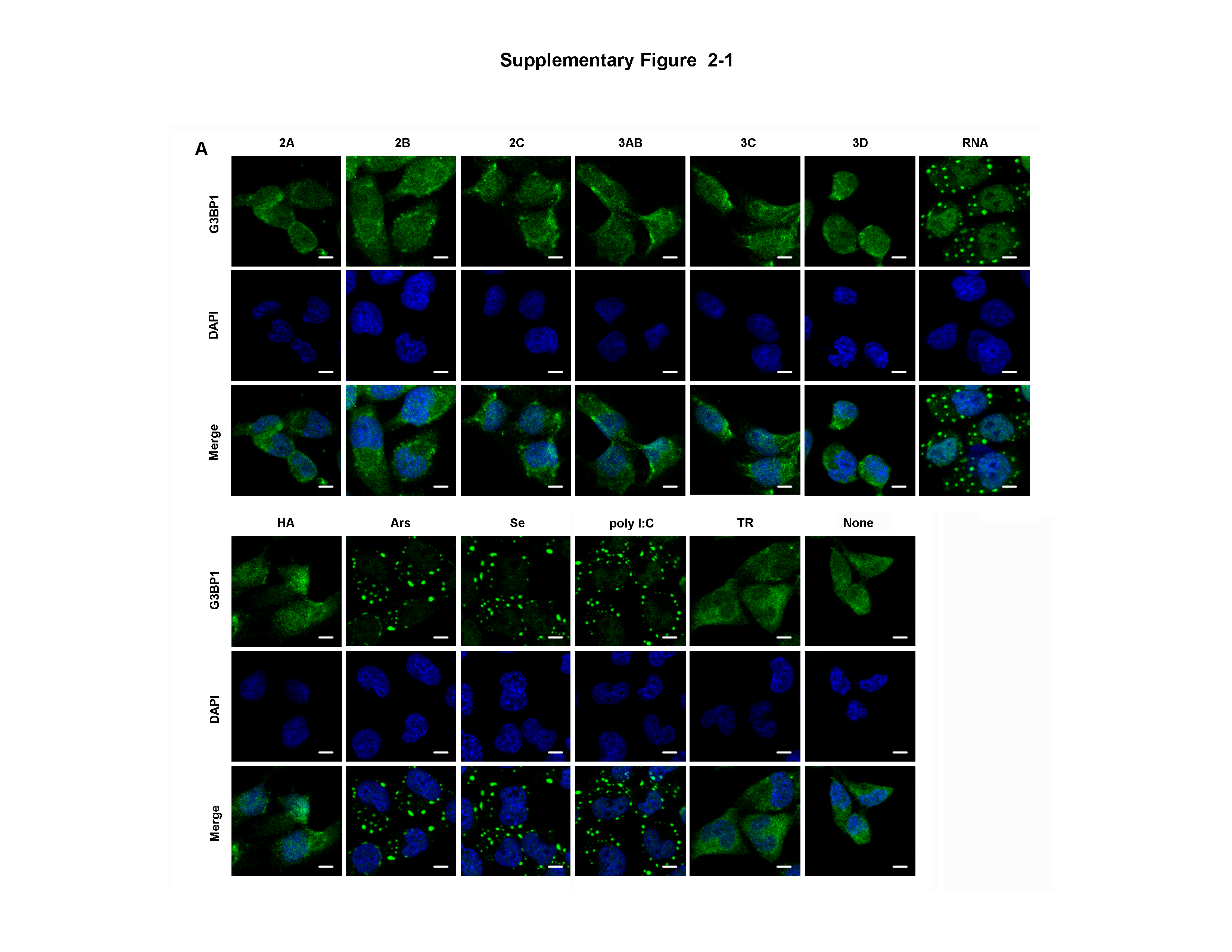

Supplement: FIGURE S1 — CA16 induced canonical SG formation. (A) RD cells were subjected to CA16 infection at an MOI of 1 or mock infection for 24 h in the presence of 50 μg/ml CHX or DMSO (Ctrl). SGs were examined by fluorescence microscopy (G3BP1 and TIA1 serve as SG markers). Representative images of stress granules are shown. Scale bars, 5 μm. (B) and (C) Quantitation of the data in (A). Graphs show the mean ± SEM, 6 random fields and 10 cells per field were examined for confocal microscopy. *p < 0.05; **p < 0.01; ***p < 0.001. [file Data_Sheet_1.zip › Image 2 A.TIF]

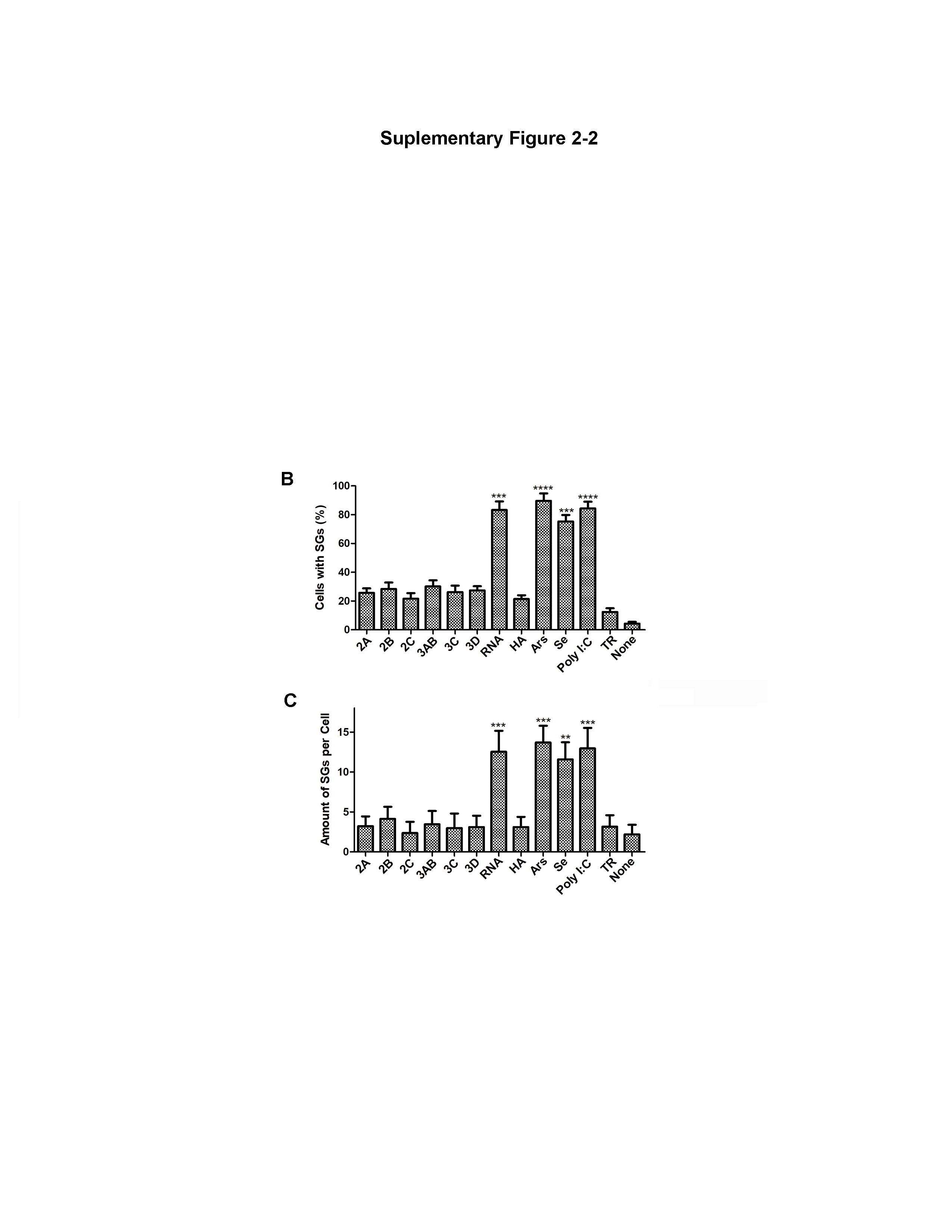

Supplement: FIGURE S1 — CA16 induced canonical SG formation. (A) RD cells were subjected to CA16 infection at an MOI of 1 or mock infection for 24 h in the presence of 50 μg/ml CHX or DMSO (Ctrl). SGs were examined by fluorescence microscopy (G3BP1 and TIA1 serve as SG markers). Representative images of stress granules are shown. Scale bars, 5 μm. (B) and (C) Quantitation of the data in (A). Graphs show the mean ± SEM, 6 random fields and 10 cells per field were examined for confocal microscopy. *p < 0.05; **p < 0.01; ***p < 0.001. [file Data_Sheet_1.zip › Image 2 B, C.TIF]

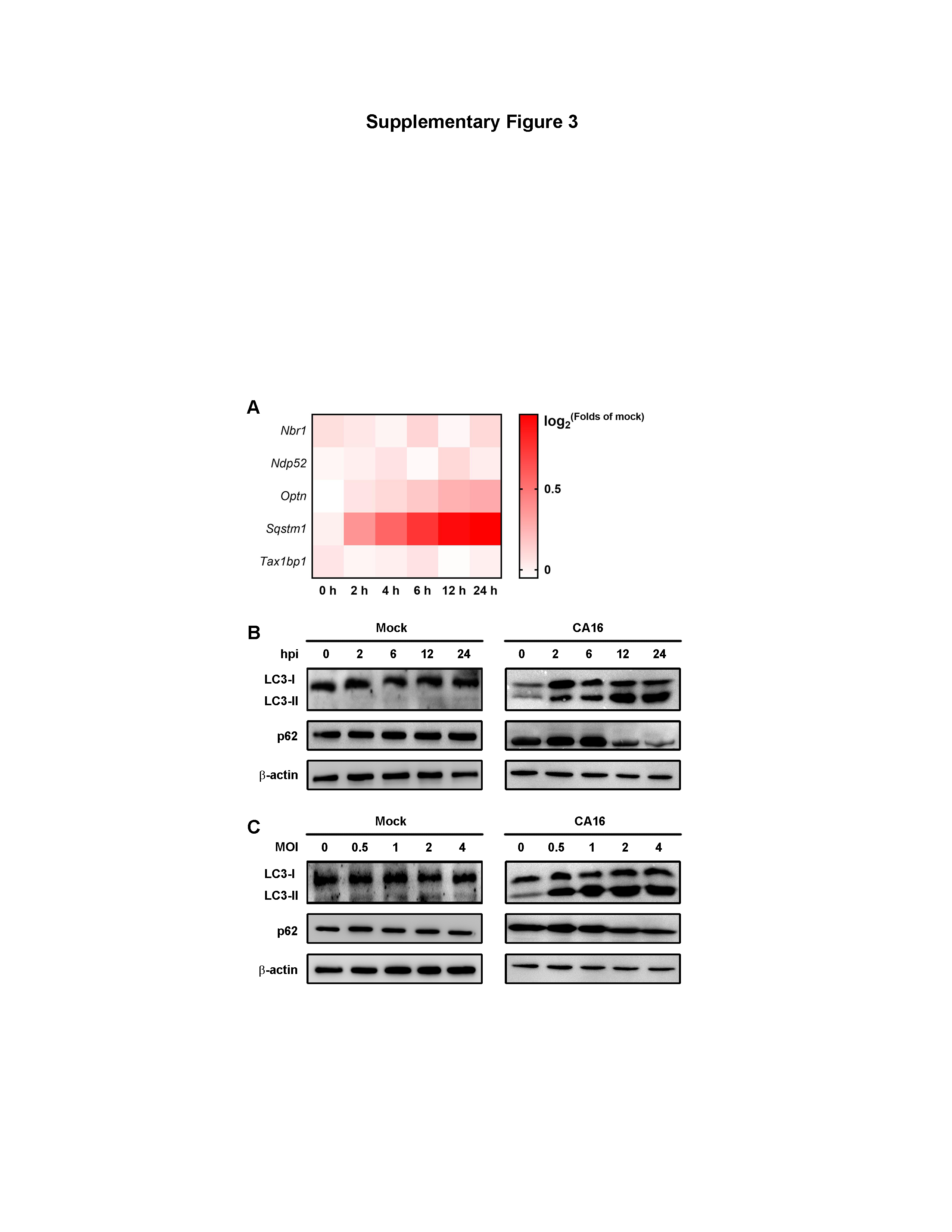

Supplement: FIGURE S1 — CA16 induced canonical SG formation. (A) RD cells were subjected to CA16 infection at an MOI of 1 or mock infection for 24 h in the presence of 50 μg/ml CHX or DMSO (Ctrl). SGs were examined by fluorescence microscopy (G3BP1 and TIA1 serve as SG markers). Representative images of stress granules are shown. Scale bars, 5 μm. (B) and (C) Quantitation of the data in (A). Graphs show the mean ± SEM, 6 random fields and 10 cells per field were examined for confocal microscopy. *p < 0.05; **p < 0.01; ***p < 0.001. [file Data_Sheet_1.zip › Image 3.TIF]

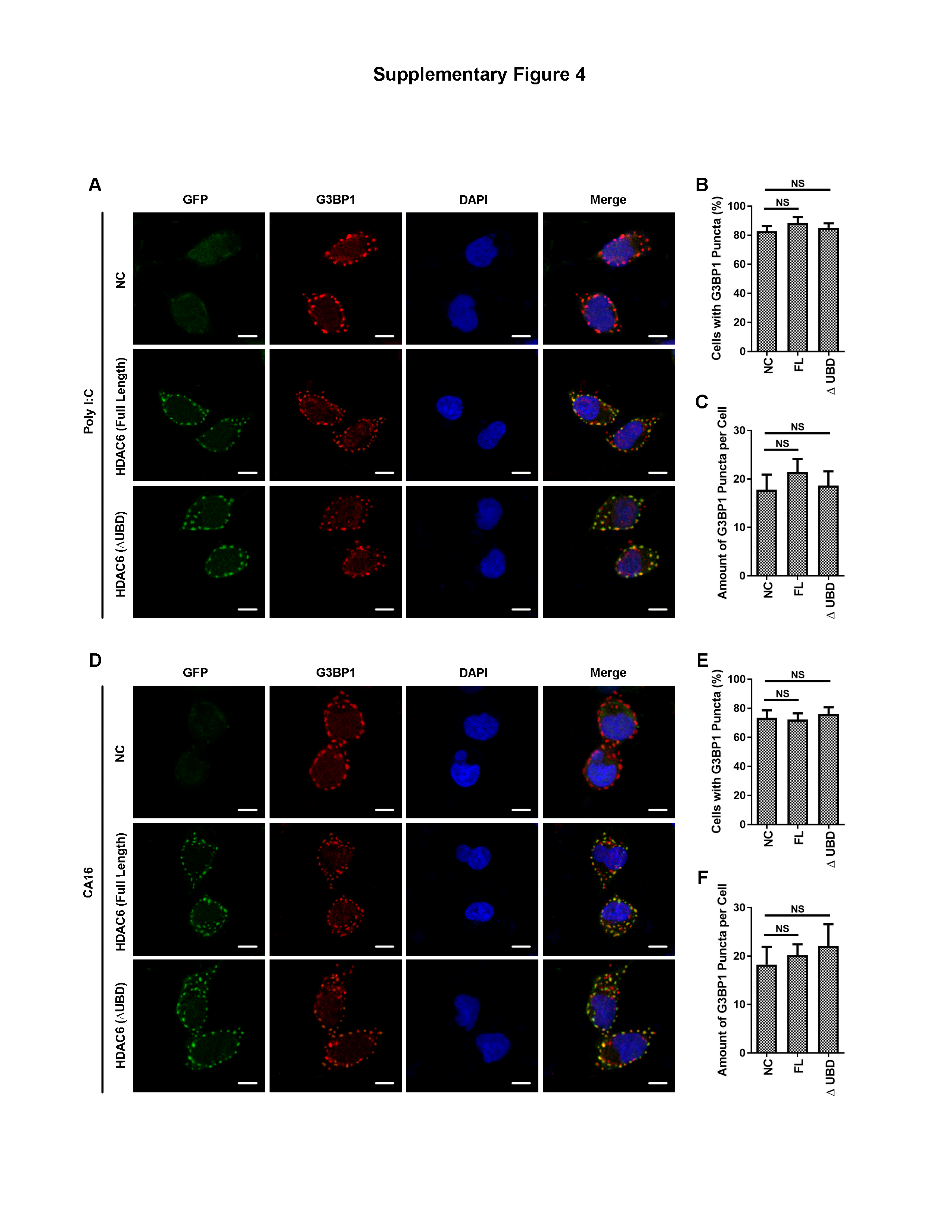

Supplement: FIGURE S1 — CA16 induced canonical SG formation. (A) RD cells were subjected to CA16 infection at an MOI of 1 or mock infection for 24 h in the presence of 50 μg/ml CHX or DMSO (Ctrl). SGs were examined by fluorescence microscopy (G3BP1 and TIA1 serve as SG markers). Representative images of stress granules are shown. Scale bars, 5 μm. (B) and (C) Quantitation of the data in (A). Graphs show the mean ± SEM, 6 random fields and 10 cells per field were examined for confocal microscopy. *p < 0.05; **p < 0.01; ***p < 0.001. [file Data_Sheet_1.zip › Image 4.TIF]

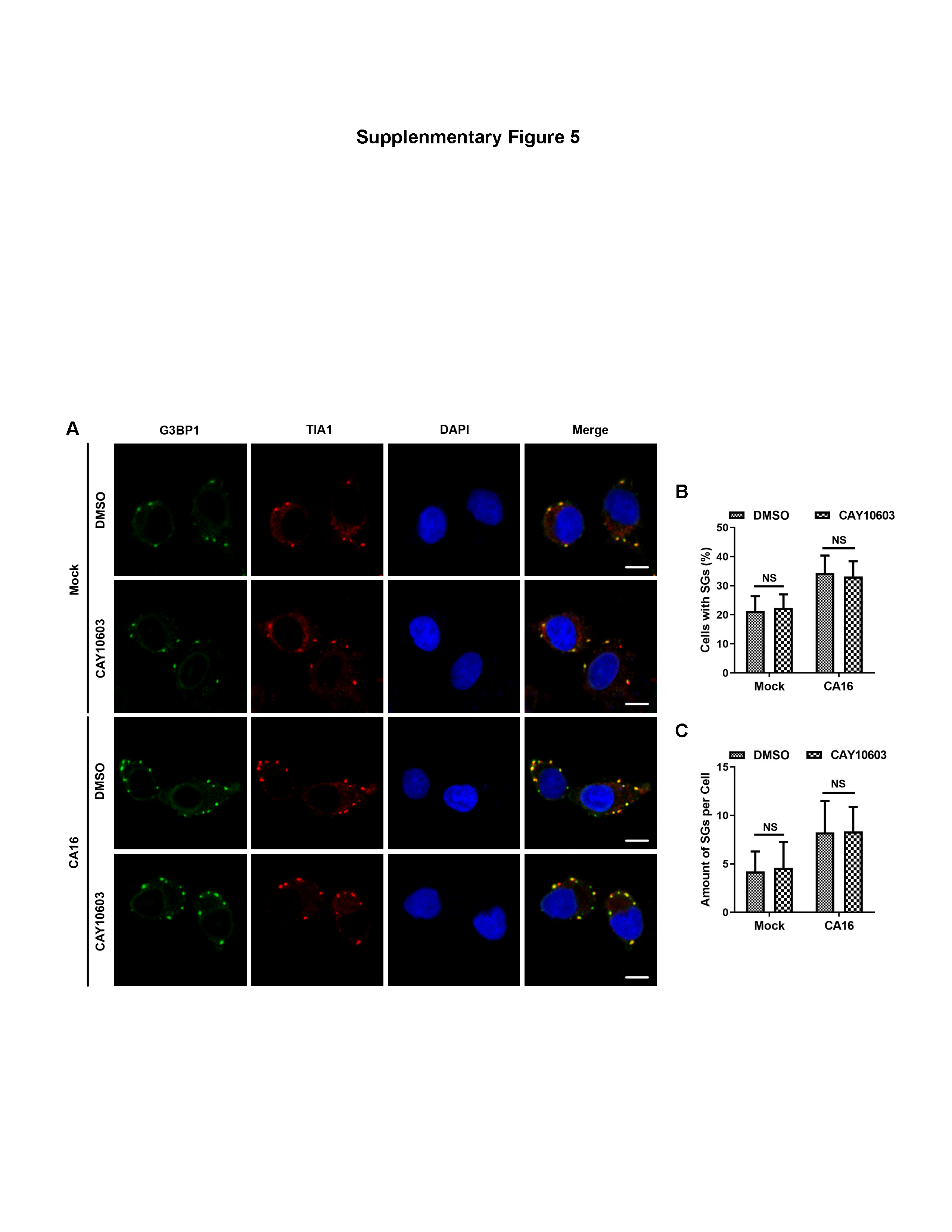

Supplement: FIGURE S1 — CA16 induced canonical SG formation. (A) RD cells were subjected to CA16 infection at an MOI of 1 or mock infection for 24 h in the presence of 50 μg/ml CHX or DMSO (Ctrl). SGs were examined by fluorescence microscopy (G3BP1 and TIA1 serve as SG markers). Representative images of stress granules are shown. Scale bars, 5 μm. (B) and (C) Quantitation of the data in (A). Graphs show the mean ± SEM, 6 random fields and 10 cells per field were examined for confocal microscopy. *p < 0.05; **p < 0.01; ***p < 0.001. [file Data_Sheet_1.zip › Image 5.TIF]

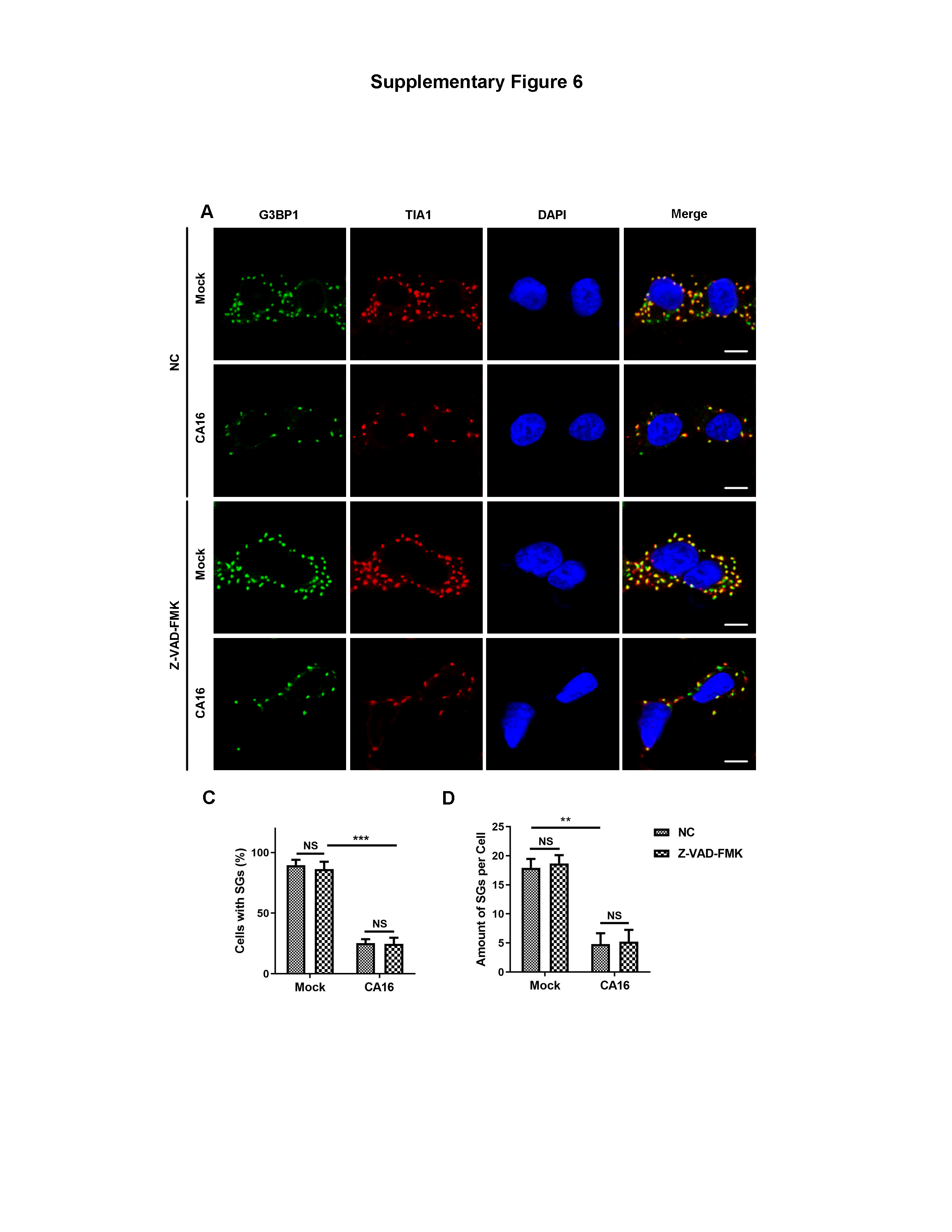

Supplement: FIGURE S1 — CA16 induced canonical SG formation. (A) RD cells were subjected to CA16 infection at an MOI of 1 or mock infection for 24 h in the presence of 50 μg/ml CHX or DMSO (Ctrl). SGs were examined by fluorescence microscopy (G3BP1 and TIA1 serve as SG markers). Representative images of stress granules are shown. Scale bars, 5 μm. (B) and (C) Quantitation of the data in (A). Graphs show the mean ± SEM, 6 random fields and 10 cells per field were examined for confocal microscopy. *p < 0.05; **p < 0.01; ***p < 0.001. [file Data_Sheet_1.zip › Image 6.TIF]
